# Case of Hydrophobia, in Which Bleeding Appears to Have Been of Service

**Published:** 1828-07

**Authors:** Charles Davis

**Affiliations:** Member of the Royal College of Surgeons in Ireland, &c.


*Case of Hydrophobia, in which Bleeding appears to have been of service.* By CHARLES DAVIS, M.D. Member of the Royal College of Surgeons in Ireland, &c.

ON Wednesday, March 19th, 1828, I was sent for by my friend, Dr. JOHN ELLIOTT, to take charge of Joseph Keating, a boy between eleven and twelve years old, who resided at Newcomen Bridge, North Strand, and was labouring under hydrophobia. I received the following account of the case: He had been several times bitten, but more especially about four months since he had received a bite from a strange dog, whose history could not be accurately made out. The injury consisted of three large and deep lacerations upon the inner and fore part of his right thigh, over the course of the saphena vein and femoral artery. For these wounds he had been received into Stevens's Hospital, where they were at first dressed, and afterwards poulticed. He remained in the hospital for sixteen days; but at the end of that period, the wounds being still open, and presenting a sloughy surface, he ran away. It appears that his mother applied poultices, smeared with oil of turpentine, to the wound; under which treatment they rapidly granulated, and ultimately healed.

On the 14th, he was observed to remain at home, instead of going to play as usual. On the 15th, his mother, in some way, spilt or threw some water upon him, on which he was much agitated, and appeared to be choked. In the course of the afternoon he was observed to be gloomy and desponding, and his eyes were red. The cicatrix of the wound on the thigh was found, on examination, to be sore, having "cracked" and given vent to some sero-sanguineous exudation. Throughout the two ensuing days, he appeared to grow worse; became gloomy, shy, and strange; screamed occasionally; ate nothing, but frequently wanted to drink, yet could not swallow.

On Wednesday, the 19th, when I entered the room, he looked at me obliquely, and with marked suspicion; his eyes were red and suffused; his cheeks had a livid flush; his respiration was hurried; his pulse very frequent; his tongue much furred. He complained of pain in his back and at the pit of the stomach; his bowels were confined, and he had difficulty in voiding his urine. He had no appearance of vesicles, or apthæ, under his tongue, nor fulness in the region of the salivary glands; but the sterno-mastoid muscles had their outline rather more defined and evident than in health, and his general susceptibility to impressions seemed increased.

I breathed gently upon his face, upon which he was instantly attacked by a violent convulsion, during which the cervical muscles were called powerfully into action.

Throughout this convulsion, he seemed to suffer very considerable constriction of the pharynx and larynx; his cheeks became suffused and livid, and his face (the mouth more especially) was very much protruded. The convulsion resembled exceedingly, but was much more violent than, the shivering spasm which occurs upon jumping into cold water, or upon receiving the shock of the cold shower-bath. Asking him if he would take some water, putting a spoon upon a plate, or pouring out water within his hearing, produced a similar convulsion.

On showing him a piece of looking-glass, he started back in the wildest terror, stretching out his arms, and became violently convulsed. The three cicatrices upon his thigh were red; the largest had upon it several cracks, which crossed it obliquely; the discharge of reddish serum had ceased, but he complained of a

"stinging" pain in them all. Touching any of them gave him considerable uneasiness; and pressure made below the cartilages of the ribs, if directed upwards, had a similar effect. He complained much of thirst. On offering him a teaspoonful of water, the sight of it produced a convulsion, but after that had subsided, he made several desperate, but unavailing, efforts to carry the spoon to his mouth.

As I had seen purgatives, narcotics, antispasmodics, and strychnine, prove utterly ineffectual in former instances, and encouraged by the success which I had in a case of tetanus, in which the patient recovered from a state as rigid and immovable as a statue, chiefly under the very liberal use of the lancet, aided by purgatives, I determined upon employing a similar plan of treatment in the present instance: in addition to which, I decided upon the removal of the cicatrices without a moment's loss of time.

I opened a vein in his right arm; but, as the blood flowed slowly, I also opened another in his left, and thus abstracted a quantity amounting to sixteen ounces or more.

The effects of this bleeding were decidedly advantageous: the eye lost much of its wildness, and the violence of the spasms was diminished; he became able to permit his arm to be bathed with warm water, which we could not have attempted before.

I now proceeded to excise the cicatrices. He complained chiefly of the lowest, which was the largest; it was red and cracked, and covered with a scaly cuticle, which was detached in some places. The removal of these cicatrices required a cautious dissection, in which I was assisted by Dr. Elliott and Mr. Richard Coote. During this operation the boy exhibited as great a degree of fortitude as I have ever seen evinced, and, indeed, his general character was that of extreme determination.

I prescribed for him—

> **Pulv. Jalapæ c. ʒj.; Calomel gr. vj.; Pulv. Zingib. gr. ix. in ch. tres quarum, capiat j. secundis horis donec, &c.**

At half-past seven in the evening, his bowels had not been opened; his pulse was 156, the frequency apparently depending much upon the convulsion; his tongue furred; the expression of his countenance wild. The noise of water poured in the next room into a vessel, brought on a violent paroxysm; and presenting him with a glass of cold water had a similar effect. After many efforts, he succeeded in swallowing a teaspoonful of warm tea.

> **Enema purgans statim.—Applic$^{r}$ Emplastrum Lyttæ epigastrio et hypochondrio dextro.—℞. Pulv. Jalapæ c. ℈iv.; Calomel gr. vj.; Pulv. Ipecac. c. gr. ix. M. div. in ch. iij. quarum, capiat j. secunda quaque hora donec, &c.**

March 20th, eight A.M.—Bowels three times moved, stools fetid and bilious; has taken but one of the powders last directed; pulse 156, rather hurried and feeble; face somewhat flushed; endeavours occasionally to get up some viscid phlegm; has much difficulty in making water; tongue much furred; skin nearly

[redacted]
natural. The testicles appear to be drawn upwards towards the rings, while the scrotum is relaxed. At times he lies quiet, with the pupil of the eye covered by the upper lid; at other times, flings his arms about impatiently; but at all times, and even while he seems inattentive, is suspiciously watchful of every one around, as was several times exemplified. The noise of the tea things in the next room produced a severe convulsion, which was reproduced in a violent degree by breathing gently in his face. Light and noise appear to distress him, and, when any one approaches him, he tosses out his arms, or else covers up his head in the bedclothes; the pupil somewhat dilated. When questioned, he answers rationally. The wounds upon his thigh do not exhibit the slightest appearance of inflammation, and, but that no blood flows from them, they look as they did immediately after they were inflicted.

Half-past seven P.M.—The blister has risen; pulse upwards of 160, not full nor strong; face flushed; respiration hurried. Has been sleeping, but awoke with fright, and said that water had been thrown on him. Delirious during the day, frequently desiring the attendants "to turn out that dog." His teeth covered with a viscid saliva; his tongue very much furred; his expression of countenance that of wild timidity; his whole appearance much changed for the worse. The least excitement brought on the spasm, and the slightest breathing upon his face threw him into a convulsion so severe that he remained for a considerable time afterwards apparently much exhausted.

Under these circumstances, although his general appearance very little invited the employment of the lancet, guided rather by the result of the previous bleeding than by the present symptoms, I bled him to the amount of eight ounces; which was followed by an obvious alleviation of the symptoms. In employing this bleeding, I considered that, as his circumstances were desperate, I was justified in recurring to the remedy which had before relieved him. I prescribed a purging enema, which brought away a bilious, green, and fetid stool; and directed that a long blister should be applied to the spine, from the occiput to the middle of the back.

The sight of a looking-glass presented to him this evening, induced a convulsion similar to the appearance which a child presents when suddenly plunged into cold water.

At nine o'clock on the morning of the 21st, I learned that, after I had left him, he had slept for some hours; that the blister had partially risen; that he had swallowed some warm tea several times throughout the night. His appearance improved; pulse under 160; tongue furred, two apthæ of a white colour are visible upon its apex. Has had one bilious stool. Appears on his guard against the effects of the noise of water, or of being asked to drink, either of which now only produce a sort of smile, in which he seems to compress his lips, and to exert a powerful command over the muscles of his face, by which he succeeds in preventing the spasm.

Dr. Harkan breathed gently upon his face while stooping to examine his tongue, which instantly brought on the convulsion. He swallowed some warm milk with a spasmodic effort, but appeared much improved. The objections of his family to a repetition of the venesection were insurmountable.

On this day he was visited by Mr. Peile, who agreed with Dr. Harkan, Dr. Harvey, Mr. Flood, and the other medical gentlemen who had seen him, that his case was exquisitely well-marked hydrophobia.

I visited him on the evening of the 21st, when I found him still further improved; and, with a view to allay the general irritability, which was present, I directed that he should take ten drops of the Acetum Opii every fourth hour.

At half-past twelve on the 22d, he appeared much better: his pulse was 126 or 130. I learned that, on the preceding evening, he had made an attempt to bite his mother; and that the convulsion had several times through the night been brought on, while some children, who lay in the room, made water. He drank some wine and warm milk, but when he was offered a few drops of whiskey in a wine-glass, the convulsion recurred with violence.

At half-past seven P.M. he appeared to have improved; his pulse about 120; states that he is better. His tongue is cleaning at the edges. He complains of the pain of the blistered parts.

As he appeared somewhat heavy and sleepy, I directed that the use of the Acetum Opii, of which he had taken twenty drops, should be stopped; and, as the sight of transparent glass vessels had the effect of reproducing the convulsion, I desired that his drink, which was to consist chiefly of mulled port wine, should be given him in a teacup.

March 23d, twelve M.—He is much improved: his pulse 108; his tongue quite clean, the two apthæ on its tip are still visible; no thirst, but an appetite for drink. Appears irritable, and scratches the blistered parts unceasingly. His bowels have been naturally open, and a vesicular eruption is observable: the vesicles are a sixth of an inch in diameter, resembling those of pemphigus, and are not very numerous; they contain a turbid serum, are not surrounded with any areola of inflammation, are chiefly observable upon the fore part of the chest, but some are scattered over the extremities.

Today, as his symptoms were considerably improved, I thought it unnecessary to direct any medicine, but desired that he should have wine and warm milk, both of which he drank with tolerable facility, and with relish. On this day, blowing upon his face produced a slight convulsion, but in every other respect he had materially amended.

On the evening of the 23d, I found him still better: his bowels had been twice moved naturally; pulse 100; tongue clean; the the aphthæ still visible; has drank freely throughout the day; the vesicular eruption still apparent.

**Inhibeatur Enema purgans.**

On the morning of the 24th, he appeared still better. I directed that he should continue the milk diet as before.

In the evening, his pulse was under 100; tongue clean; appetite for drink considerable.

**Enema purgans statim.**

As I had lately met with considerable advantage from the Ammoniuret of Copper as a tonic and antispasmodic, in some cases of chorea which occurred in children who were patients at Saint Thomas's Dispensary, and deeming the exhibition of a tonic likely to accelerate his recovery, of which I entertained hopes, I prescribed for him a fourth of a grain of that medicine every fourth hour.

25th, twelve M.—No hydrophobic symptoms apparent; tongue rather dry; pulse 100, somewhat hard; respiration somewhat hurried and quick; short cough occasionally, and expectoration of viscid mucus; bowels confined; urine scanty and high coloured. I directed that the use of the Ammoniuret of Copper, of which he had taken not more than three-fourths of a grain, should be discontinued.

**Capiat Pulv. Jalapæ c. ℈j.; Calomel gr. ij.; Pulv. Zingib. gr. iij. secundis horis.—Enema purgans post horas iv.**

—and I left instructions that venesection should be freely practised, if the inflammatory symptoms continued after the bowels should be opened.

This morning I was unexpectedly called away out of town, and, upon my return the next night, I found the boy dying of pneumonia. It appeared that the symptoms of inflammation in the lungs had progressively increased; that his cough, unaccompanied with expectoration, had become frequent and distressing; his pulse hard and full; his tongue dry; his chest greatly oppressed. The stethoscope had clearly indicated the existence of pneumonia at the posterior and inferior part of both lungs, the *ralê crepitante* being audible at the corresponding parts of the chest, while the *ralê puerile* was heard throughout the remainder, and the *ralê muqueuse* was very evident. The abdomen also was somewhat tender under pressure.

Notwithstanding these symptoms, and every argument which had been employed to convince her to the contrary, his mother had obstinately resisted the further abstraction of blood, either by the lancet or by leeches; neither would she permit the application of a blister, nor allow him to take medicine of any sort. He died on the evening of the 26th, the fourteenth day from the setting-in of the disease.

In fact, as the opinion which I had given of the result had been unfavorable at the commencement, the friends of the boy, from their own idea of the nature of this disease, esteemed every exertion rather in the light of an experiment, than as likely to be followed by benefit.

Sixteen hours after his death, I took the opportunity of making a dissection, in presence of the Bishop-street pupils. Within the cranium and spinal canal, every part was healthy, except that the pia mater in both of these regions seemed rather more florid than usual; but the difference, if real, was extremely trivial. The appearance of the pharynx, œsophagus, and stomach were perfectly natural. Both lungs, at the inferior and posterior part, presented the traces of high inflammation, being violet coloured and indurated. A quantity of reddish serum issued from a section of them when squeezed, and from several distinct points blood and pus was observed to flow. The liver was occupied throughout by the small brown tubercle; its size was diminished, and the gallbladder was greatly reduced in magnitude. A small portion of the jejunum was intussuscepted, and the part which was inclosed within the lower portion was somewhat diminished in its calibre. The serous surface of the neighbouring portion of jejunum was rather more vascular than usual, but the intussusception appeared to be very recent. The descending colon was slightly contracted in its calibre, as if by the action of its circular fibres; and, at the sigmoid flexure, a considerable degree of contraction, approaching to stricture, and apparently from a similar cause, existed for the extent of an inch. The rectum was empty and contracted; the bladder also was empty, and had contracted very closely. Every other part was healthy.

Just before his death, his mother had been prevailed upon to permit the abstraction of blood from his arm, but at a period when the measure was hopeless. The blood, which was drawn at this time, differed much from that which had previously been taken away, as its coagulum was firm and resisting, cupped upon its surface, and would no doubt have presented the buffy coat, had it flowed more rapidly from the vein; while the blood which had been drawn during the continuance of the hydrophobic symptoms was flat upon its surface, and loosely coagulated, as we usually find it to be in diseases attended with spasmodic action of the muscular system.

Although little analogy appears to exist between hydrophobia, which is a febrile and infectious disease, and tetanus, which is uninfectious and not necessarily accompanied with fever, yet it is worthy of remark, that, in the instance of Patrick Higgins, to whose case of tetanus I have above alluded, the patient narrowly escaped falling a victim to pneumonia, at a time when the tetanic symptoms were nearly at an end; and I may also observe, that, in all those cases of hydrophobia which have fallen under my observation, the patients, shortly before the setting-in of the symptoms, had been exposed to some considerable excitement.

