# Errata

**Published:** 1828-08

**Authors:** 


					ERRATA.-
-The line at the top of p. 11 should have been placed at the bottom of p. 12.?

				

